# Lipoteichoic acid restrains macrophage senescence via β‐catenin/FOXO1/REDD1 pathway in age‐related osteoporosis

**DOI:** 10.1111/acel.14072

**Published:** 2023-12-21

**Authors:** Weike Cheng, Yong Fu, Zexin Lin, Mouzhang Huang, Yingqi Chen, Yanjun Hu, Qingrong Lin, Bin Yu, Guanqiao Liu

**Affiliations:** ^1^ Department of Orthopaedics Nanfang Hospital, Southern Medical University Guangzhou China; ^2^ Guangdong Provincial Key Laboratory of Bone and Cartilage Regenerative Medicine Nanfang Hospital, Southern Medical University Guangzhou China

**Keywords:** FOXO1, macrophage senescence, mTOR, osteoporosis, REDD1, β‐Catenin

## Abstract

Osteoporosis and its related fractures are common causes of morbidity and mortality in older adults, but its underlying molecular and cellular mechanisms remain largely unknown. In this study, we found that lipoteichoic acid (LTA) treatment could ameliorate age‐related bone degeneration and attenuate intramedullary macrophage senescence. FOXO1 signaling, which was downregulated and deactivated in aging macrophages, played a key role in the process. Blocking FOXO1 signaling caused decreased REDD1 expression and increased phosphorylation level of mTOR, a major driver of aging, as well as aggravated bone loss and deteriorated macrophage senescence. Moreover, LTA elevated FOXO1 signaling through β‐catenin pathway while β‐catenin inhibition significantly suppressed FOXO1 signaling, promoted senescence‐related protein expression, and accelerated bone degeneration and macrophage senescence. Our findings indicated that β‐catenin/FOXO1/REDD1 signaling plays a physiologically significant role that protecting macrophages from senescence during aging.

## Graphical abstract

Osteoporosis and its related fractures are common causes of morbidity and mortality in older adults. We found LTA prevents the downregulation of β‐catenin/FOXO1/REDD1 signaling pathway and upregulation of p‐mTOR with aging, inhibiting the macrophage senescence and bone degeneration. This study establishes the essential role of β‐catenin/FOXO1/REDD1 signaling pathway in bone degeneration during aging.

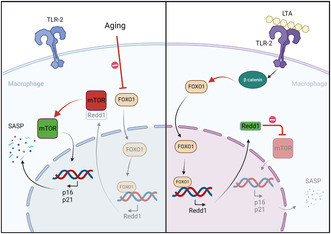

**Abbreviations**
ANOVA: Analysis of variance
BMDM: Bone marrow–derived macrophage
BMSCs: Bone marrow‐derived mesenchymal stem cells
BV/TV: Percent bone volume
Ct. Ar: Cortical bone area
Ct. Th: Cortical bone thickness
DDIT4: DNA damage inducible transcript 4
DIS: drug‐induced senescent
FBS: Fetal bovine serum
FOXO1: Forkhead box O1
GAPDH: Glyceraldehyde‐3‐phosphate dehydrogenase
HSCs: Hepatic stellate cells
IL‐1: Interleukin‐1
IL‐6: Interleukin‐6
LTA: Lipoteichoic acid
mTOR: Mammalian target of rapamycin
PBS: Phosphate‐buffered saline
PCNA: Proliferating cell nuclear antigen
PCR: Polymerase chain reaction
PRR: Pattern recognition receptor
RANKL: Receptor activator of nuclear factor‐κ B ligand
REDD1: Regulated in development and DNA damage response 1
ROS: Reactive oxygen species
RPMI: Roswell Park Memorial Institute medium
SASP: Senescence‐associated secretory phenotype
SA‐β‐Gal: Senescence‐associated β‐galactosidase
Tb. N: Trabecular number
Tb. Sp: Trabecular separation
Tb. Th: Trabecular thickness
TLR‐2: Toll‐like receptor‐2
TNF‐α: Tumor necrosis factor‐α
μCT: Microcomputed tomography

## 1 INTRODUCTION

Osteoporosis is characterized by deterioration of bone microarchitecture and density, along with an increase in fragility fracture, causing morbidity, mortality, and worse quality of life (Wang, Fang, et al., 2023). Recent findings have pointed out that cellular senescence, always described as the irreversible cell cycle arrest, apoptosis resistance, and senescence‐associated secretory phenotype (SASP) (Liu, Chai, et al., 2021), is closely associated with the development of age‐related chronic diseases, including osteoporosis. Eliminating these senescent cells in bone significantly prevented age‐related bone loss (Yu & Wang, 2022). Moreover, senescent macrophages accumulated in the bone marrow could release grancalcin and SASP to induce skeletal aging (Li et al., 2021). Inhibition of the related signaling pathways could suppress the senescence of macrophages so as to promote bone health. Therefore, senescent macrophages may play a crucial role in the development of osteoporosis. Lipoteichoic acid (LTA), a component in the cell wall of gram‐positive bacteria, is relevant to cellular senescence regulation. LTA could significantly decrease the SASP secretion and improve the general appearance of aging mice, as well as reduce the ROS production of fibroblasts and prevent the photoaging of skin (Hong et al., 2015). Furthermore, LTA also accelerated bone formation in mouse femoral defect model (Hu et al., 2020). Nevertheless, the effect of LTA on osteoporosis is rarely reported.

The Wnt/β‐catenin signaling pathway is a highly conserved pathway that participates in multiple aspects of cellular function. In particular, the expression and distribution of β‐catenin in different organs vary according to age (García‐Velázquez & Arias, 2017). Activation of β‐catenin, which is downregulated in aging bone (García‐Velázquez & Arias, 2017), inhibits osteoclastogenesis and prevents osteoporosis (Cui et al., 2022). The β‐catenin signaling has also been reported to be regulated in cellular senescence. Recent study demonstrated that Wnt/β‐catenin signaling was repressed at the early stage of cellular senescence (Ye et al., 2007). Blocking β‐catenin signaling by inhibiting the β‐catenin nuclear translocation could induce cell senescence, and restoring β‐catenin signaling could reduce p53 Ser15 phosphorylation and prevent cell senescence (Wang et al., 2021). Furthermore, it has been reported that β‐catenin can bind to and activate FOXO1 to promote its downstream signal transcription (Qiao et al., 2018). FOXOs, the Forkhead proteins, are transcription factors that have consistently been regarded as the “longevity genes”. There are four FOXO genes: FOXO1, FOXO3, FOXO4, and FOXO6 in mammals. FOXO1, as a major transcription factor of FOXOs family, is abundantly expressed in bone tissue (Chen et al., 2021). Recently, it was reported that FOXO1 protected cells from senescence (Delpoux et al., 2021). Targeting miR‐182, a repressor of FOXO1, could restore bone formation during bone aging (Kim et al., 2012). These studies strongly suggest that β‐catenin/FOXO1 signaling might be linked to the prevention of cellular senescence and regulation of bone metabolism.

mTOR, a serine/threonine kinase of the PI3‐K–related family, is a component of two structurally and functionally distinct complexes, mTOR complex 1 (mTORC1) and mTOR complex 2 (mTORC2) (Mossmann et al., 2018). The phosphorylation level of mTOR is regarded as an important driver of aging, especially the phosphorylation of mTORC1, which accelerates cell senescence and tissue aging through the accumulation of damaged proteins and organelles, exhaustion of stem cell pools, and other aspects (Liu & Sabatini, 2020). Inhibition of mTOR signaling has been shown to delay aging, suppress the senescence‐associated secretory phenotype (SASP), and prolong life span (Herranz et al., 2015). The effect of mTOR signaling on skeletal development has been widely uncovered. mTOR signaling is reported to be associated with bone loss, and mTOR inhibition significantly increases bone mass (Spencer et al., 2006). REDD1 (Regulated in Development and DNA Damage response 1), encoded by the DDIT4 gene, is known as an endogenous mTOR inhibitor. Recent studies found REDD1 expression reduced in joint synovium, meniscus, and cartilage during aging (Alvarez‐Garcia et al., 2016, 2017). Additionally, the REDD1 promoter, which contained a FOXO1‐binding site, was activated by FOXO1 overexpression, but silenced in FOXO1^−/−^ skeletal muscle (Oyabu et al., 2022). Nevertheless, the mTOR signaling was inhibited by FOXO1 in skeletal muscle (Oyabu et al., 2022). These studies suggest that REDD1/mTOR signaling could be important in bone metabolism during aging through the regulation of FOXO1.

In the present study, downregulated β‐catenin/FOXO1/REDD1 signaling and activated mTOR signaling in macrophages were found in bone marrow during aging. LTA treatment could significantly upregulate β‐catenin/FOXO1/REDD1 signaling and inhibit mTOR signaling in macrophages, as well as prevent macrophage senescence and ameliorate bone loss in middle‐aged mice. Finally, we demonstrated that inhibition of β‐catenin/FOXO1 signaling accelerated the macrophage senescence and promoted bone degeneration in middle‐aged mice.

## 2 MATERIALS AND METHODS

### 2.1 Animals and treatments

All animal protocols were approved by the Institutional Animal Care and Use Committee at the Southern Medical University Nanfang Hospital before the study, and all animal experiments were performed in accordance with Institutional Animal Care and Use Committee guidelines. Two and 12‐month‐old C57BL/6J male mice were obtained from the Experimental Animal Center at Southern Medical University in Guangzhou, China. Mice were housed in the facility under specific pathogen‐free conditions at 25°C with a 12‐h light/dark cycle and had access to food and water ad libitum. Twelve months old male C57BL/6J mice were randomly assigned to four groups: vehicle, LTA, LTA‐AS1842856, and LTA‐IWR‐1, treated with phosphate‐buffered saline (PBS), LTA (Sigma‐Aldrich, 1.5 mg/kg, intraperitoneal injection, twice per week), LTA + AS1842856 (MCE, HY‐100596, 30 mg/kg, oral gavage, twice per week), and LTA + IWR‐1 (MCE, HY‐12238, 5 mg/kg, intraperitoneal injection, twice per week), respectively, for 2 months. After euthanasia, their femurs and tibias were dissected for further analysis.

### 2.2 Microcomputed tomography analysis

For microcomputed tomography (μCT) analysis, femurs obtained from mice were dissected free of soft tissue, fixed overnight in 70% ethanol, and analyzed by a high‐resolution μCT80 (Scanco Medical, Wangen‐Bruttisellen, Switzerland) at 7 μm resolution. We reconstructed and analyzed images using Image Processing Language v.5.15 software (Scanco Medical). The parameters, including percent bone volume (BV/TV), the trabecular thickness (Tb. Th), the trabecular number (Tb. N), the trabecular separation (Tb. Sp), the cortical bone thickness (Ct. Th), and the cortical bone area (Ct. Ar) were calculated.

### 2.3 Senescence‐associated β‐galactosidase (SA‐β‐Gal) staining and immunofluorescence staining of bone tissue sections

Mice femora were fixed overnight in 10% formalin at 4 °C for 24 h, decalcified in 0.5 M EDTA (pH 7.4) at 4 °C with constant shaking for 2 weeks and then dehydrated in 20% sucrose and 2% polyvinylpyrrolidone solution for 24 h. Finally, the tissues were embedded in OCT, and 10‐μm‐thick, longitudinally oriented bone sections were collected for staining. Senescent cells were detected using a SA‐β‐Gal staining kit (9860; Cell Signaling Technology, USA) according to the manufacturer's instructions. For immunofluorescence staining, we incubated the sections with primary antibodies to F4/80 (14–4801‐82; Invitrogen, USA), β‐catenin (8480; Cell Signaling Technology, USA), FOXO1 (18592‐1‐AP; Proteintech, China), REDD1 (10638‐1‐AP; Proteintech), and phospho‐mTOR^ser2448^ (67778‐1‐Ig; Proteintech) overnight at 4 °C, followed by incubation with Rhodamine (TRITC)–conjugated Goat Anti‐Rat IgG (H + L; SA00007‐7; Proteintech), Fluorescein (FITC)–conjugated Affinipure Goat Anti‐Rabbit IgG (H+L; SA00003‐2; Proteintech), or Fluorescein (FITC)–conjugated Affinipure Goat Anti‐Mouse IgG (H+L; SA00003‐1; Proteintech) secondary antibodies. Nuclei were counterstained with DAPI (Solarbio). All sections were observed under a Ti2‐E microscope (Nikon, Tokyo, Japan). For simultaneous SA‐β‐Gal staining and immunofluorescence staining, sections were first subjected to a β‐Gal staining followed by immunofluorescence staining as described above. To obtain the colocalization image, an immunofluorescence imaging was obtained first followed by a brightfield imaging of the same field.

### 2.4 Bone marrow‐derived macrophage (BMDM) culture

BMDM was obtained from 8‐week‐old male mice bone marrow following the protocol as previously described (Assouvie et al., 2018). Briefly, the bilateral femurs and tibias were separated and free of soft tissues. After both ends of long bones were removed, the bone marrow was eluted out with Roswell Park Memorial Institute medium (RPMI) 1640 (Gibco, USA), 2% fetal bovine serum (FBS; Gibco, USA), and penicillin/streptomycin (p/s). After filtered and centrifuged, the pellet was washed using RPMI‐1640 with 10% FBS, resuspended in differentiation medium: RPMI‐1640 (Gibco, USA), with 10% FBS, 20% L929 conditional medium, 1% p/s, and cultured at 37°C in a 5% CO_2_ incubator. The medium was replaced on day 3. Cells were harvested and transferred to six‐well plates (5 × 10^5^ cells/ well) on day 5 and cultured in differentiation medium for further 2 days. L929 cells were cultured in low‐glucose Dulbecco's modified Eagle's medium, with 10% FBS and 1% p/s in a humidified incubator at 5% CO_2_, 37°C for 7 days. The supernatant was collected, filtered, and stored at −20°C before using.

### 2.5 Cell models

As described previously (Jiang et al., 2020) a drug‐induced senescent (DIS) cell model was established, 50 μM hydrogen peroxide (H_2_O_2_) was applied to stimulate mature BMDM for 24 h. Cells were pretreated with 10 μM IWR‐1 (MCE) or 1 μM AS1842856 (MCE) for 30 min before the addition of 1 μg/mL LTA. On day 8, total RNA and protein were harvested, and expression of the genes was detected using real‐time quantitative PCR and Western blotting, respectively.

### 2.6 SA‐β‐Gal staining and immunofluorescence staining of BMDMs

Once the DIS cell model was established, senescent cells were detected by a SA‐β‐Gal staining kit (9860; Cell Signaling Technology, USA) following the manufacturer's instructions. For immunofluorescence staining, we incubated the cells with primary antibodies to FOXO1 (18592‐1‐AP; Proteintech, China) overnight at 4 °C, followed by incubation with Fluorescein (FITC)–conjugated Affinipure Goat Anti‐Rabbit IgG (H+L; SA00003‐2; Proteintech) secondary antibodies. Nuclei were counterstained with DAPI (Solarbio).

### 2.7 Western blotting

Total cell lysates were extracted in RIPA lysis buffer supplemented by protease/phosphatase inhibitor cocktail (KGP2100; KeyGEN BioTECH, Nanjing, China) and incubated for 30 min on ice. The supernatant was collected after centrifugation. The nuclear and cytoplasmic lysates were extracted by using a nuclear and cytoplasmic extraction kit (KGBSP00; KeyGEN BioTECH, Nanjing, China) following the manufacturer's protocol. The protein concentrations were determined by PierceTM BCA Protein Assay Kit (SE248347; Thermo Fisher Scientific). 30 μg of protein from each sample was separated by SDS‐PAGE and transferred to PVDF membranes. The membranes were incubated with the primary antibodies, such as β‐catenin (8480; Cell Signaling Technology), FOXO1 (18592‐1‐AP; Proteintech), phospho‐FOXO1^Ser256^ (9461; Cell Signaling Technology), REDD1 (10638‐1‐AP; Proteintech), mTOR (67778‐1‐Ig; Proteintech), phospho‐mTOR^ser2448^ (67778‐1‐Ig; Proteintech), GAPDH (10494‐1‐AP; Proteintech), and PCNA (13,110; Cell Signaling Technology), at 4°C overnight and subsequently with the secondary antibody conjugated with horseradish peroxidase at room temperature for 1 h. Signals were developed by ECL (MilliporeSigma, Burlington, MA, USA) and visualized by chemiluminescence apparatus. Protein densitometry was quantified by ImageJ software (National Institutes of Health, Bethesda, MD, USA).

### 2.8 RNA isolation and real‐time quantitative PCR analysis

Total RNA was extracted from cells using RNA extraction kit (Accurate Biology, AG21024) according to the manufacturer's protocol. Complementary DNA (cDNA) was prepared using the cDNA Synthesis SuperMix for qPCR (YEASEN, 11141ES60). Real‐time quantitative PCR was performed using SYBR GreenMaster Mix (YEASEN, 11184ES50). The primer sequence for the genes used in this study were as follows: GAPDH (forward, 5′‐TGTCGTGGAGTCTACTGGTG‐3′; reverse, 5′‐GCATTGCTGACAATCTTGAG‐3′), p16^INK4a^ (forward, 5′‐GAAAGAGTTCGGGGCGTTG‐3′; reverse, 5′‐GAGAGCCATCTGGAGCAGCAT‐3′), p21 (forward, 5′‐GTGGGTGTCAAAGCACTTAG‐3′; reverse, 5′‐ACAGTCCAGACCAGGATGTTA‐3′), p53 (forward, 5′‐ATCGCCTTCGACATCATCGC‐3′; reverse, 5′‐CCCCATGCGTACTCCATGAG‐3′), FOXO1 (forward, 5′‐ CTACGAGTGGATGGTGAAGAGC‐3′; reverse, 5′‐CCAGTTCCTTCATTCTGCACTCG‐3′), and REDD1 (forward, 5′‐ CAAGGCAAGAGCTGCCATAG‐3′; reverse, 5′‐CCGGTACTTAGCGTCAGGG‐3′). The relative expression of each target gene was calculated using 2^−ΔΔCT^ method with GAPDH for normalization.

### 2.9 Statistics

Data were presented as means ± standard errors. Unpaired Student *t*tests were used for comparisons between two groups. For multiple comparisons, one‐way analysis of variance (ANOVA) with Tukey's post hoc test was used. All data were normally distributed and had similar variation between groups. *p* < 0.05 was considered significant. All representative images of bones or cells were selected from at least three independent experiments.

## 3 RESULTS

### 3.1 LTA ameliorates age‐related osteoporosis and attenuates intramedullary macrophage senescence

To determine whether LTA might regulate osteoporosis in age‐related mice, we started with a contrastive study in middle‐aged 12‐month‐old mice treated with vehicle (vehicle group) versus 12‐month‐old mice treated with LTA (LTA group). Femora were harvested and the microstructure was measured by μCT (Figure S1a). We found that the BV/TV (*p* < 0.01), Tb. Th (*p* < 0.05), and Tb. N (*p* < 0.001) were significantly higher but Tb. Sp was significantly lower in the LTA group, compared with the vehicle group (Figure S1b–e). There was no significant alteration in the Ct. Th and Ct. Ar (Figure S1f,g). These results indicate that LTA significantly improves trabecular bone mass in 12‐month‐old mice.

As the causal role of senescent cells in age‐associated bone loss is widely known, we examined whether LTA increased trabecular bone mass through attenuating senescent cells. SA‐β‐Gal staining presented significantly fewer senescent cells in bone marrow in the LTA group, compared with the vehicle group (Figure S1h,i), indicating that LTA attenuated intramedullary cellular senescence.

**FIGURE 1:**
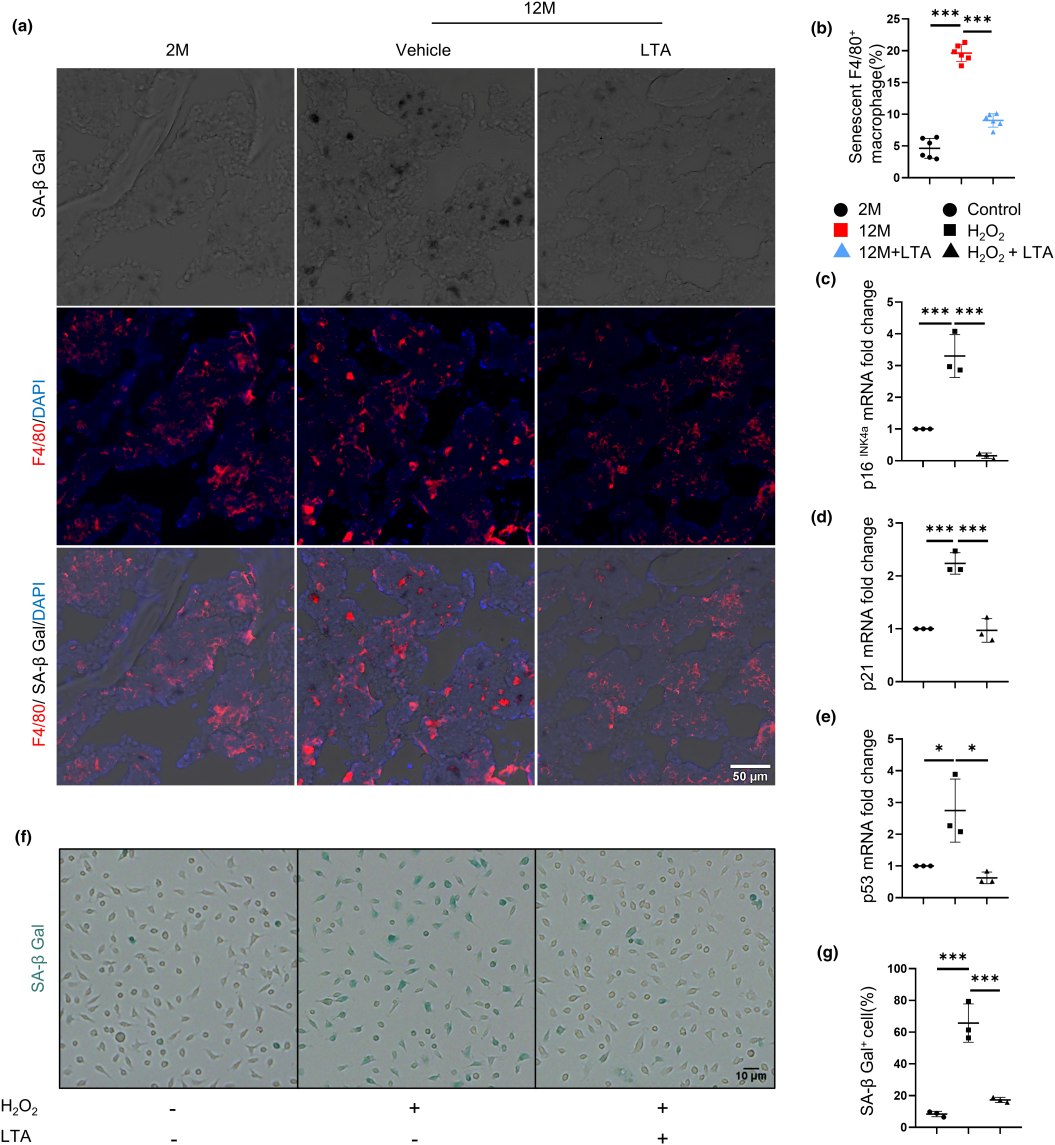
LTA rescues macrophage senescence. Representative images (a) of SA‐β‐Gal staining (gray) and immunofluorescence staining of F4/80 (red) of femoral sections from vehicle‐treated 2‐month‐old mice, vehicle‐ and LTA‐treated 12‐month‐old mice, respectively. Quantification (b) of the number of senescent F4/80^+^ macrophages in femoral bone. *n* = 6/group. Scale bars, 50 μm. Quantitative real‐time PCR analysis of p16^INK4a^ (c), p21 (d), and p53 (e) for BMDMs cultured with H_2_O_2_ together with LTA. *n* = 3/group. Representative images of SA‐β‐Gal staining (blue) (f) and quantification (g) of the percentage of SA‐β‐Gal^+^ BMDMs for three groups of BMDMs. *n* = 3/group. Scale bars, 10 μm. *p < 0.05, **p < 0.01, ***p < 0.001. Data are presented as mean ± SD. One‐way ANOVA with Tukey's test.

Previous studies have proved that senescent immune cells, especially macrophages, can accumulate in the bone marrow to induce skeletal aging (Li et al., 2021). LTA could inhibit cell senescence in the kidney, liver, and thymus (Yi et al., 2009). It still needs to be clarified whether LTA might be associated with macrophage senescence during aging. In the present study, we found that the number of senescent F4/80^+^ macrophages was significantly increased in the vehicle group and decreased dramatically in the LTA group (Figure 1a,b), indicating that LTA could restrain macrophage senescence during age‐related osteoporosis.

H_2_O_2_ has been widely used to mimic the chronic accumulation of ROS to induce cellular senescence (Kim et al., 2022). In vitro, we treated bone marrow‐derived macrophages (BMDMs) with H_2_O_2_ in the presence or absence of LTA. Results showed that LTA could offset the elevation mRNA expression of senescence‐associated genes (p16^INK4a^, p21, and p53) caused by H_2_O_2_ (Figure 1c–e). Consistently, LTA significantly suppressed the increased ratio of SA‐β‐Gal^+^ BMDMs induced by H_2_O_2_ (Figure 1f,g). These findings suggest that LTA rescues macrophage senescence and prevents bone loss in age‐related mice.

### 3.2 LTA prevents macrophage senescence by inhibiting FOXO1 downregulation and deactivation

**FIGURE 2:**
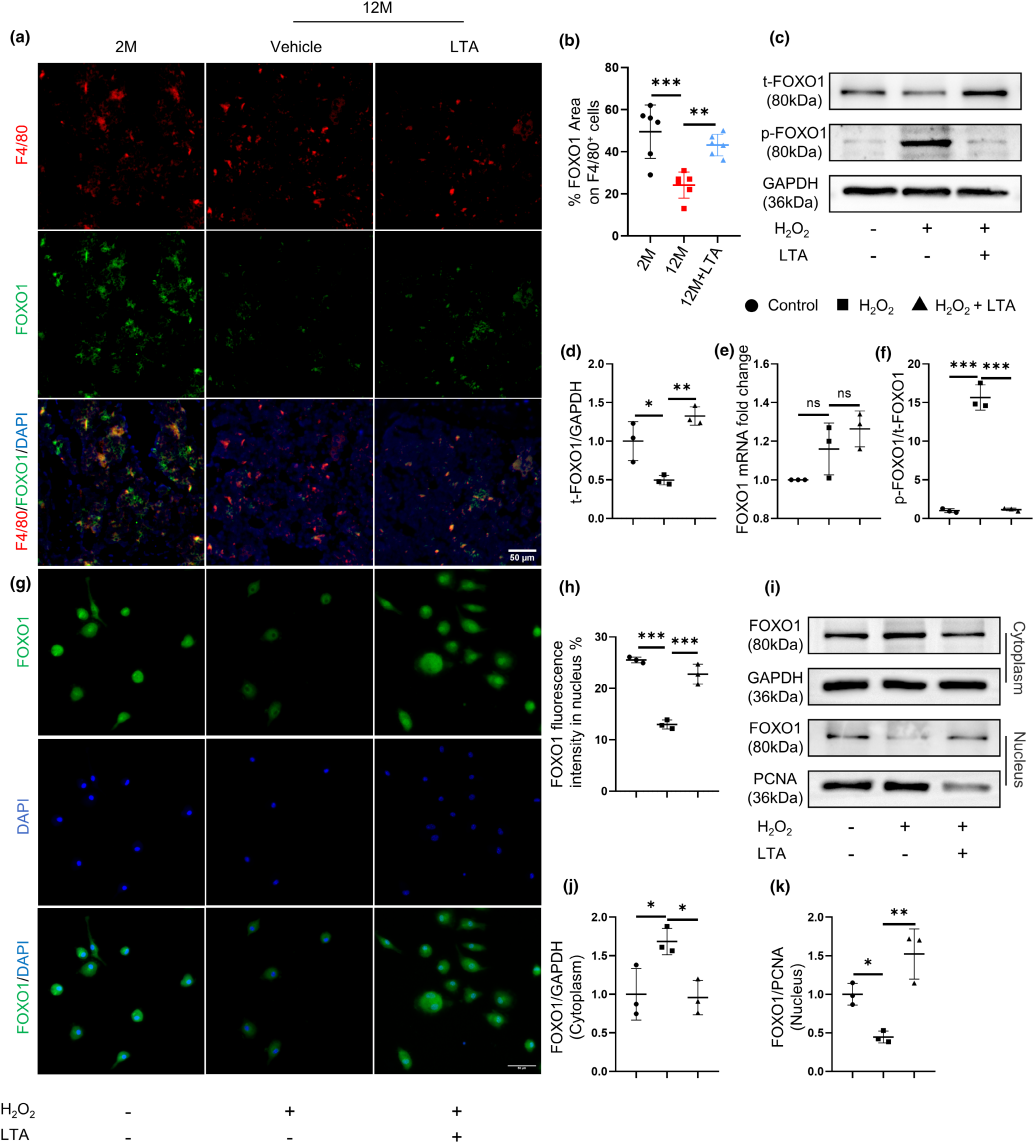
LTA prevents senescence‐induced FOXO1 downregulation and deactivation. Representative images (a) of double‐immunofluorescence staining of F4/80 (red) and FOXO1 (green) and quantification (b) of the percentage of FOXO1 expression area on F4/80^+^ cells in femoral bone. *n* = 6/group. Scale bars, 50 μm. BMDMs were treated with H_2_O_2_ together with LTA. Representative images (c) and quantification of Western blot of the relative intensity of total FOXO1 (d) and phosphorylated FOXO1 (f). *n* = 3/group. Quantitative real‐time PCR analysis of FOXO1 (e) for three groups of BMDMs. *n* = 3/group. Representative images (g) and quantification (h) of immunofluorescence staining of nuclear FOXO1 in three groups of BMDMs. *n* = 3/group. Scale bars, 50 μm. Representative images (i) and quantification of Western blot of the relative intensity of cytoplasmic FOXO1 (j) and nuclear FOXO1 (k) in three groups of BMDMs. *n* = 3/group. *p < 0.05, **p < 0.01, ***p < 0.001. Data are presented as mean ± SD. One‐way ANOVA with Tukey's test.

FOXO1, as a major transcription factor of the forkhead O (FOXOs) family, is abundantly expressed in bone tissue and stimulates bone formation (Chen et al., 2021). It is reported that FOXO1 participates in the regulation of cell senescence by regulating apoptosis, energy metabolism, DNA repair, etc (Salih & Brunet, 2008). The expression of FOXO1 was significantly decreased in the liver (Tomobe et al., 2013) and aorta (Zhu et al., 2021) of SAMP8 aging mice and normal aging mice, respectively. We next tested whether FOXO1 expression might change in macrophages during aging. Double‐immunofluorescence staining showed significantly declined FOXO1 expression in F4/80^+^ macrophages in 12‐month‐old mice compared with 2‐month‐old mice, but LTA treatment elevated FOXO1 expression in F4/80^+^ macrophages in 12‐month‐old mice (Figure 2a,b). Then, we examined the protein expression of FOXO1 in BMDMs by Western blot analysis. LTA offset the downregulated expression of FOXO1 induced by H_2_O_2_ (Figure 2c,d). Therefore, LTA prevents the downregulation of FOXO1 during senescence.

Interestingly, no significant alteration of FOXO1 mRNA level was observed in the process (Figure 2e). We hypothesized that the changes of FOXO1 protein level were due to post‐translational modifications, including phosphorylation and dephosphorylation (Kamoshita et al., 2022). The transcriptional function of FOXO1 is regulated by phosphorylation, and the nuclear localization of dephosphorylated FOXO1 is a key step in stimulating the downstream target gene transcription (Zhang et al., 2021). Hence, we evaluated the expression of phosphorylated FOXO1 (p‐FOXO1), as well as the nuclear and cytoplasmic FOXO1 in BMDMs by Western blot analysis and immunofluorescence staining. We found that the p‐FOXO1 level increased in the H_2_O_2_ group, whereas LTA suppressed the phosphorylation of FOXO1 (Figure 2c,f). Furthermore, H_2_O_2_ significantly increased the cytoplasm FOXO1 expression and inhibited the nuclear FOXO1 expression. LTA restored FOXO1 expression in nuclear but suppressed cytoplasm FOXO1 expression (Figure 2g–k). These results strongly suggest that LTA treatment suppresses FOXO1 phosphorylation and promotes FOXO1 nuclear translocation inhibited by H_2_O_2_‐induced senescence.

We further investigated whether inhibition of FOXO1 signaling might promote the senescence of macrophages in bone and accelerate bone degeneration. AS1842856, a specific FOXO1 inhibitor, was used in the LTA‐treated 12‐month‐old mice. We found that blocking FOXO1 signaling significantly reduced the BV/TV (*p* < 0.01), Tb. Th (*p* < 0.01), and Tb. N (*p* < 0.05), but increased Tb. Sp (*p* < 0.05) in the LTA‐treated 12‐month‐old mice (Figure S2a–e). There was no significant alteration in the Ct. Th and Ct. Ar (Figure S2f,g). These results indicated that inhibiting FOXO1 could promote age‐associated osteoporosis. Next, we determined if blocking FOXO1 signaling would enhance macrophage senescence. The results showed an elevated number of senescent F4/80^+^ macrophages when FOXO1 signaling was blocked in bone (Figure S2h,i). Meanwhile, AS1842856 significantly reversed the downregulated p16^INK4a^, p21, and p53 mRNA expression caused by LTA in BMDMs (Figure S2j–l). Consistently, we observed increased senescent BMDMs by AS1842856 treatment (Figure S2m,n). These observations indicate that LTA prevents the downregulation of FOXO1 to ameliorate bone degeneration and macrophage senescence during aging.

### 3.3 LTA inhibits mTOR phosphorylation through FOXO1/REDD1 signaling pathway

**FIGURE 3:**
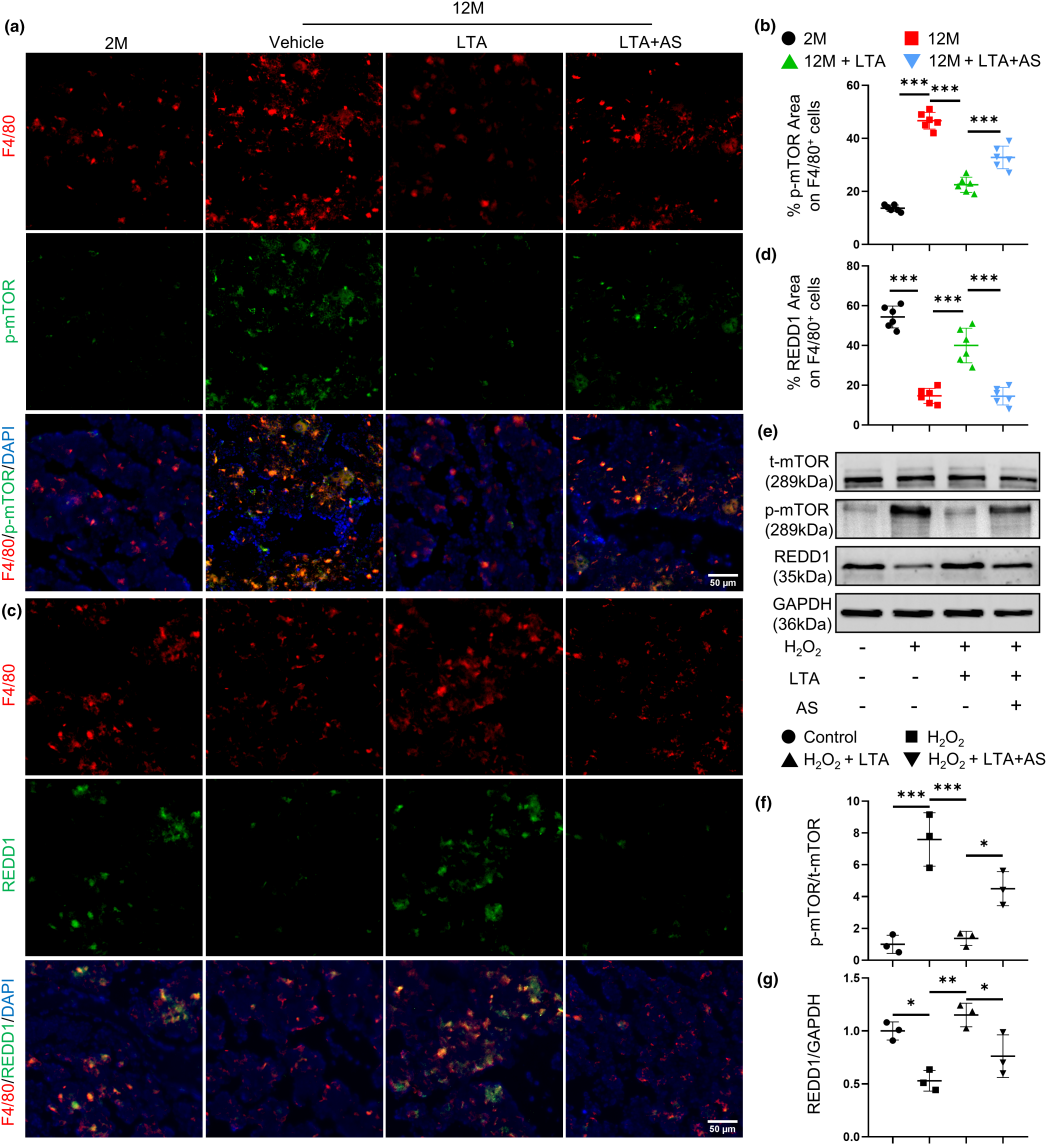
LTA inhibits mTOR phosphorylation through FOXO1/REDD1 signaling pathway. Representative images (a) of double‐immunofluorescence staining of F4/80 (red) and p‐mTOR (green) and quantification (b) of the percentage of p‐mTOR expression area on F4/80^+^ cells in femoral bone of vehicle‐treated 2‐month‐old mice, vehicle‐treated 12‐month‐old mice and LTA‐treated 12‐month‐old mice in the presence or absence of AS. *n* = 6/group. Scale bars, 50 μm. Representative images (c) of double‐immunofluorescence staining of F4/80 (red) and REDD1 (green) and quantification (d) of the percentage of REDD1 expression area on F4/80^+^ cells in the femoral bone of four groups of mice. *n* = 6/group. Scale bars, 50 μm. BMDMs were treated with H_2_O_2_ together with LTA and AS. Representative images (e) and quantification of Western blot of the relative intensity of p‐mTOR (f) and REDD1 (g) in four groups of BMDMs. n = 3/group. AS: AS1842856. *p < 0.05, **p < 0.01, ***p < 0.001. Data are presented as mean ± SD. One‐way ANOVA with Tukey's test.

The mTOR activation is tightly related to cellular senescence (Harrison et al., 2009) and bone loss (Liu & Sabatini, 2020; Moriceau et al., 2010; Spencer et al., 2006). We first detected the phosphorylation level of mTOR in 2‐month‐old and 12‐month‐old mice. Double‐immunofluorescence staining showed significantly upregulated p‐mTOR expression in F4/80^+^ macrophages in the bone of 12‐month‐old mice compared with 2‐month‐old mice, which was inhibited by LTA treatment (Figure 3a,b). Besides, LTA could offset the elevated expression of p‐mTOR in BMDMs induced by H_2_O_2_, as shown in Western blotting results (Figure 3e,f).

REDD1, as an endogenous mTOR inhibitor, has been reported to decline in aging joint synovium, meniscus, and cartilage (Alvarez‐Garcia et al., 2016, 2017). We further detected the REDD1 expression in the bone of 2‐month‐old and 12‐month‐old mice. Downregulated REDD1 expression in F4/80^+^ macrophages was observed during aging, which could be restored by LTA (Figure 3c,d). LTA also rescued the declined REDD1 mRNA level (Figure S3) and protein level (Figure 3e,g) in BMDMs caused by H_2_O_2_. These results suggested that macrophages express less REDD1 thus enhancing the phosphorylation of mTOR in bone during aging. LTA restores the REDD1 expression and inhibits p‐mTOR expression.

REDD1 is one of the downstream target genes transcripted by FOXO1 (Oyabu et al., 2022). To test whether the alteration of REDD1/p‐mTOR signaling might be ascribed to FOXO1, we further performed immunofluorescence staining. Results showed that AS1842856 significantly upregulated p‐mTOR and downregulated REDD1 expression in F4/80^+^ macrophages (Figure 3a–d) in LTA‐treated 12‐month‐old mice. Blocking FOXO1 signaling in BMDMs showed increased expression level of p‐mTOR and decreased expression level of REDD1 (Figure 3e–g). These data indicate that LTA inhibits the phosphorylation of mTOR through FOXO1/REDD1 signaling pathway.

### 3.4 Elevation of β‐catenin induced by LTA regulates cellular senescence through FOXO1/REDD1/mTOR signaling pathway

**FIGURE 4:**
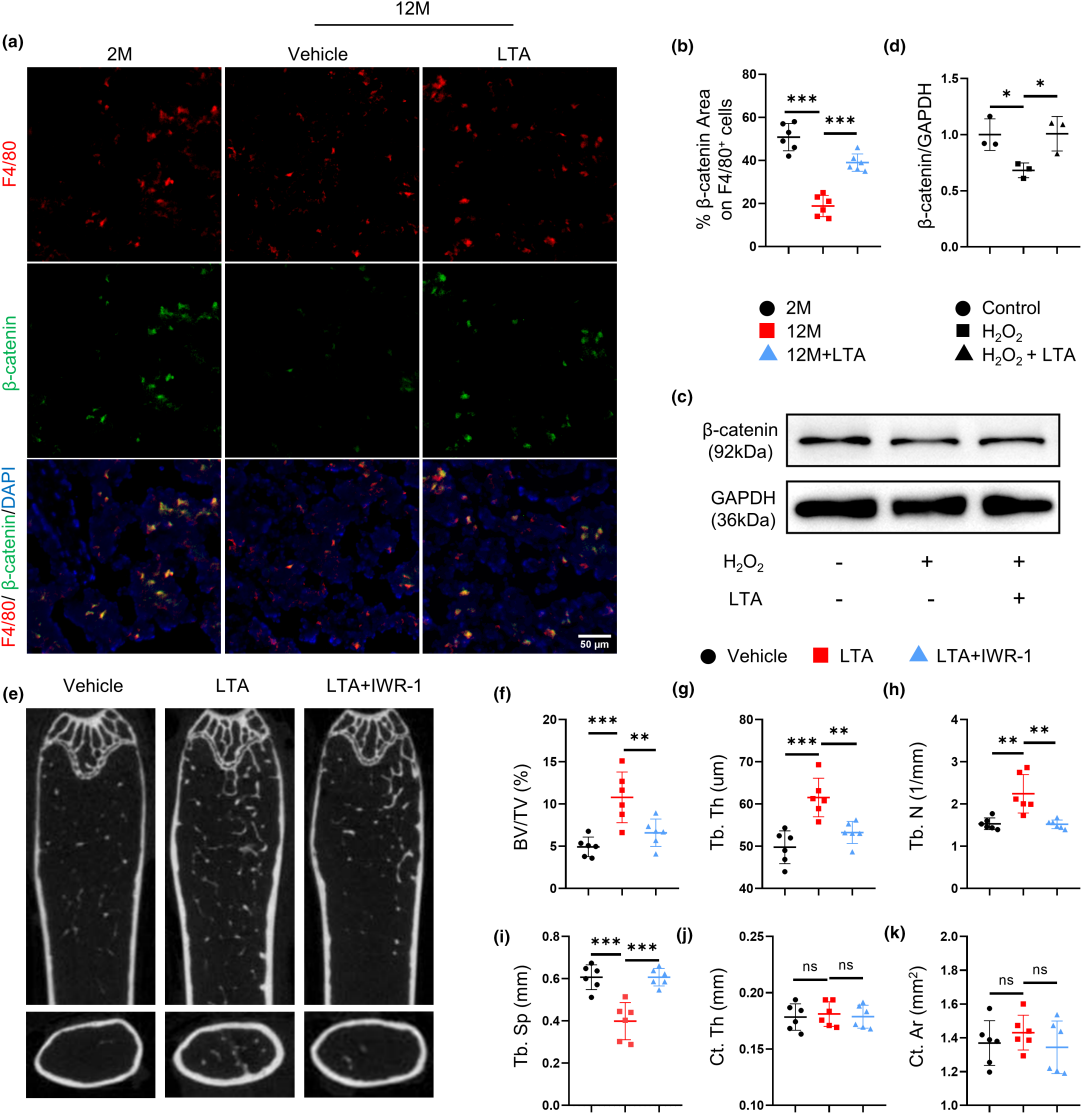
LTA prevents the downregulation of β‐catenin during senescence to ameliorate bone degeneration. Representative images (a) of double‐immunofluorescence staining of F4/80 (red) and β‐catenin (green) and quantification (b) of the percentage of β‐catenin expression area on F4/80^+^ cells in the femoral bone of vehicle‐treated 2‐month‐old mice, vehicle‐treated 12‐month‐old mice and LTA‐treated 12‐month‐old mice. *n* = 6/group. Scale bars, 50 μm. BMDMs were treated with H_2_O_2_ together with LTA. Representative images (c) and quantification of Western blot of the relative intensity of β‐catenin (d) in three groups of BMDMs. *n* = 3/group. 12‐month‐old mice were treated with LTA together with IWR‐1. Representative μCT images (e) of longitudinal sections and cross sections of femoral bone and quantitative analyses of trabecular bone volume fraction (BV/TV) (f), trabecular thickness (Tb. Th) (g), trabecular number (Tb. N) (h), and trabecular separation (Tb. Sp) (i), cortical bone thickness (Ct. Th) (j) and cortical bone area (Ct. Ar) (k); *n* = 6/group. *p < 0.05, **p < 0.01, ***p < 0.001. Data are presented as mean ± SD. One‐way ANOVA with Tukey's test.

The β‐catenin signaling is closely bounded to senescence (Cui et al., 2022). During aging, the β‐catenin signaling pathway is significantly downregulated in the brain, bone, and thymus (García‐Velázquez & Arias, 2017). We verified whether the expression level of β‐catenin might be affected in bone during aging. Interestingly, double‐immunofluorescence staining showed significantly downregulated β‐catenin expression in F4/80^+^ macrophages in 12‐month‐old mice compared with 2‐month‐old mice, but LTA treatment exhibited elevated β‐catenin expression in F4/80^+^ macrophages in 12‐month‐old mice (Figure 4a,b). Western blotting results confirmed that the expression of β‐catenin decreased significantly in BMDMs treated with H_2_O_2_ compared with BMDMs treated with vehicle, and the expression of β‐catenin recovered significantly in the present of LTA (Figure 4c,d). Thus, LTA prevents the downregulation of β‐catenin during senescence.

To investigate whether age‐related osteoporosis was linked to β‐catenin downregulation, we treated 12‐month‐old mice with LTA, in the presence or absence of IWR‐1, a β‐catenin inhibitor, for 2 months. BV/TV, Tb. Th, and Tb. N significantly decreased in IWR‐1‐treated mice, with Tb. Sp increased (Figure 4e–i), whereas no changes were found in the Ct. Th and Ct. Ar (Figure 4j,k). The results suggest LTA prevents osteoporosis through upregulating β‐catenin expression.

Meanwhile, elevated number of senescent F4/80^+^ macrophages was observed when β‐catenin signaling was blocked (Figure S4a,b). IWR‐1 also significantly promoted the downregulated p16^INK4a^, p21, and p53 mRNA expression induced by LTA (Figure S4c–e). Consistently, we observed increased senescent BMDMs by IWR‐1 treatment (Figure S4f,g). These data indicate that downregulated β‐catenin signaling in macrophages induces cell senescence.

**FIGURE 5:**
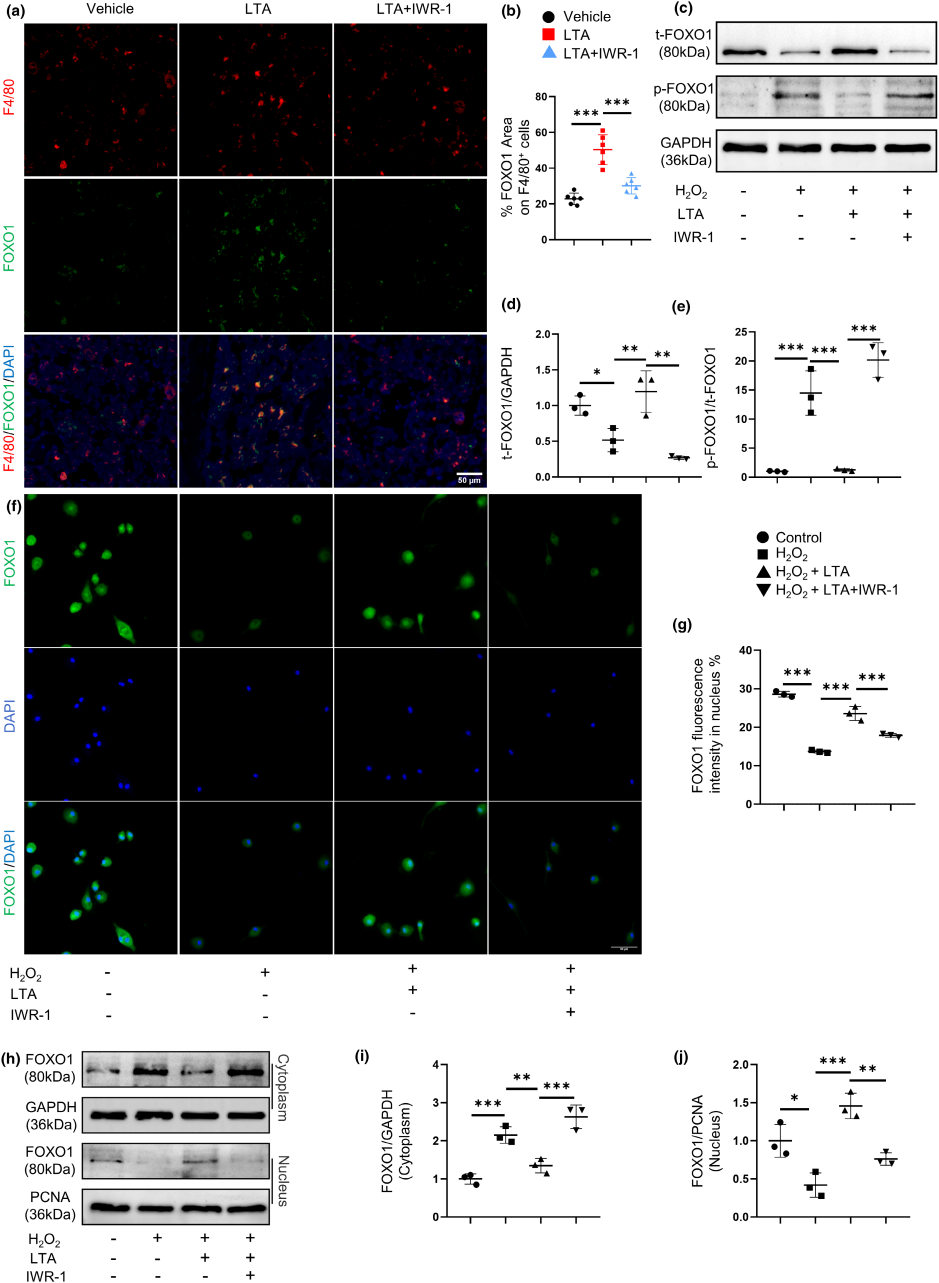
LTA stimulates FOXO1 expression and promotes FOXO1 nuclear translocation through β‐catenin. Representative images (a) of double‐immunofluorescence staining of F4/80 (red) and FOXO1 (green) and quantification (b) of the percentage of FOXO1 expression area on F4/80^+^ cells in the femoral bone of three groups of 12‐month‐old mice. *n* = 6/group. Scale bars, 50 μm. BMDMs were treated with H_2_O_2_ together with LTA and IWR‐1. Representative images (c) and quantification of Western blot of the relative intensity of total FOXO1 (d) and phosphorylated FOXO1 (e) in four groups of BMDMs. *n* = 3/group. Representative images (f) and quantification (g) of immunofluorescence staining of nuclear FOXO1 in four groups of BMDMs. *n* = 3/group. Scale bars, 50 μm. Representative images (h) and quantification of Western blot of the relative intensity of cytoplasmic FOXO1 (i) and nuclear FOXO1 (j) in four groups of BMDMs. *n* = 3/group. *p < 0.05, **p < 0.01, ***p < 0.001. Data are presented as mean ± SD. One‐way ANOVA with Tukey's test.

It has been reported that β‐catenin could bind to and activate FOXO1 to promote its downstream signal transcription (García‐Velázquez & Arias, 2017; Qiao et al., 2018). We found IWR‐1 significantly inhibited FOXO1 expression in F4/80^+^ macrophages in LTA‐treated 12‐month‐old mice (Figure 5a,b), and restrained the FOXO1 but promoted the p‐FOXO1 expression in BMDMs (Figure 5c–e). Furthermore, IWR‐1 significantly upregulated the cytoplasm FOXO1 expression but downregulated the nuclear FOXO1 expression, as shown in immunofluorescence staining (Figure 5f,g) and Western blot results (Figure 5h–j). These results suggest that LTA stimulates FOXO1 expression and promotes FOXO1 nuclear translocation through β‐catenin signaling.

**FIGURE 6:**
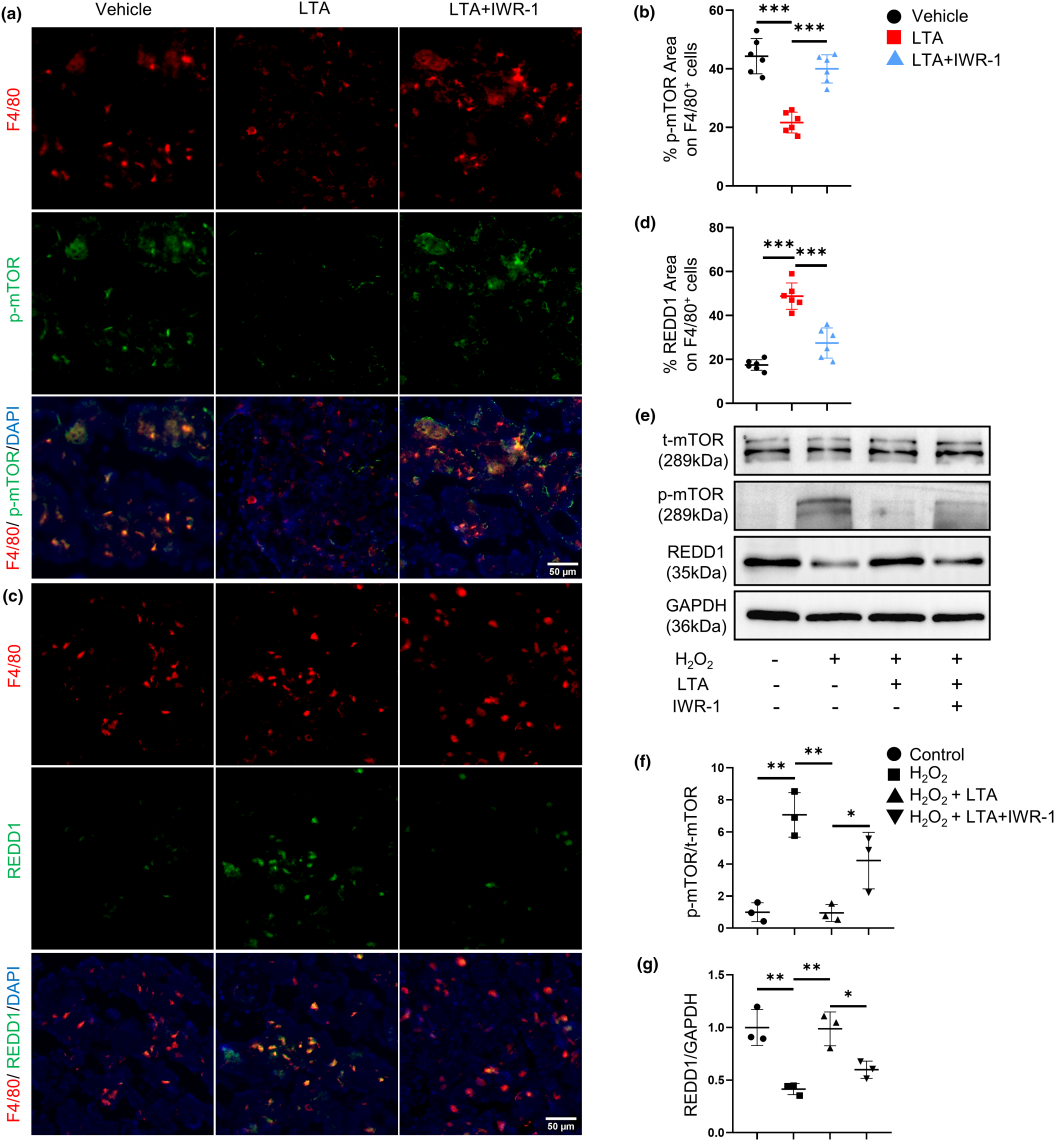
LTA inhibits mTOR phosphorylation through the β‐catenin/REDD1 signaling pathway. Representative images (a) of double‐immunofluorescence staining of F4/80 (red) and p‐mTOR (green) and quantification (b) of the percentage of p‐mTOR expression area on F4/80^+^ cells in the femoral bone of three groups of 12‐month‐old mice. *n* = 6/group. Scale bars, 50 μm. Representative images (c) of double‐immunofluorescence staining of F4/80 (red) and REDD1 (green) and quantification (d) of the percentage of REDD1 expression area on F4/80^+^ cells in the femoral bone of three groups of 12‐month‐old mice. *n* = 6/group. Scale bars, 50 μm. BMDMs were treated with H_2_O_2_ together with LTA and IWR‐1. Representative images (e) and quantification of Western blot of the relative intensity of p‐mTOR (f) and REDD1 (g) in four groups of BMDMs. *n* = 3/group. *p < 0.05, **p < 0.01, ***p < 0.001. Data are presented as mean ± SD. One‐way ANOVA with Tukey's test.

Then, we observed increased p‐mTOR (Figure 6a,b) and decreased REDD1 expression (Figure 6c,d) in F4/80^+^ macrophages when β‐catenin was blocked in LTA‐treated 12‐month‐old mice. Likewise, inhibiting β‐catenin significantly facilitated phosphorylation of mTOR but reduced REDD1 expression in BMDMs (Figure 6e–g). The data above indicate that the elevation of β‐catenin induced by LTA regulates cellular senescence through FOXO1/REDD1/mTOR signaling pathway.

## 4 DISCUSSION

Although the negative regulation of bone microarchitecture and density by senescent cells was previously reported (Liu, Chai, et al., 2021; Yu & Wang, 2022), the underlying molecular mechanisms are largely unknown. Here, we provide evidence that LTA treatment rescues downregulated β‐catenin/FOXO1/REDD1 signaling in macrophages during aging to prevent senescence of macrophages and bone degeneration, which could be offset by blocking β‐catenin/FOXO1/REDD1 signaling. Thus, our work uncovers a new cellular and molecular mechanism for the deleterious effects of senescent macrophages on bone health, and targeting the downregulated β‐catenin/FOXO1/REDD1 signaling may provide new therapeutic prevention of bone degeneration.

“Inflammaging,” also known as age‐related multiorgan chronic inflammation, is a fundamental hallmark of aging. During aging, the bone marrow microenvironment turns to be proinflammatory, which may influence the interactions between immune cells and bone cells, to mediate bone metabolism and age‐related bone loss (Yu & Wang, 2022). One of the main age‐related outcomes is the accumulation of senescent cells, which can cause detrimental effects via secreting numerous SASP. Senescent macrophages play an unignorable role in this procedure. There is a significant increase in the expression of SASP proinflammatory factors (Il‐1α, Il‐6, and TNF‐α) in senescent macrophages, which profoundly cause systemic bone loss (Yu & Wang, 2022; Zhang et al., 2021). Besides, increased proinflammatory cytokines effectively activate RANKL signaling, essential for osteoclastogenesis and subsequent bone resorption (Lam et al., 2000). Along with the secretion of SASP, senescent macrophages also release grancalcin to induce bone aging (Li et al., 2021). Furthermore, macrophages play an essential role in eliminating senescent cells, senescent macrophages with impaired function in which process likely contribute to impaired bone regeneration. A recent study reported that clearance of senescent cells in old mice presented higher bone mass and strength and better bone microarchitecture compared to vehicle‐treated mice (Farr et al., 2017). LTA is relevant to cellular senescence regulation, such as inhibiting cell senescence in the kidney, liver, and thymus (Yi et al., 2009). LTA also accelerated bone formation in mouse femoral defect model (Hu et al., 2020). Our present work further demonstrated that LTA could ameliorate macrophage senescence and promote bone health in middle‐aged mice. Although LTA has also been reported to induce senescence (Loo et al., 2017), we believe the different administration of LTA causes the difference in outcomes. In the aforementioned study, the administration of LTA was relatively high, both in vivo and in vitro. 200 μL of a solution of 5 mg/mL (equally 1 mg) LTA was used every day at 22 weeks old C57BL6 mice for 3 weeks to induce senescence. We treated C57BL6 mice with 1.5 mg/kg (equally 50 μg) LTA twice per week at the age of 12 months old for 2 months and found senescent macrophages in bone reduced. Similarly, the expression of SASP factors in HSCs were induced by LTA at the least level of 10 μg/mL in the aforementioned study. In our work, we treated BMDMs with 1 μg/mL LTA and found that the relatively low dose of LTA could prevent BMDM senescence. The inference can be supported by another study that 4 mg/kg (equally 80 μg) of LTA administration once a week for 7 weeks in mice (20 ± 2 g) presented downregulated p16 expression in multiple organs (Yi et al., 2009).

The β‐catenin signaling has been reported to be important for osteogenic differentiation in osteoporosis. Inhibition of β‐catenin signaling by miR‐320a treatment induces downregulated osteogenic differentiation (Wang et al., 2020). Likewise, inhibition of β‐catenin in osteoblasts leads to high bone resorption, low bone mass, and finally osteopenia (Huang et al., 2019). Stabilization of β‐catenin in differentiated osteoblasts negatively regulates osteoclast differentiation by favoring the expression of osteoprotegerin, resulting in high bone mass (Glass et al., 2005). In aging bones, the expression of β‐catenin signaling is downregulated (García‐Velázquez & Arias, 2017). Toll‐like receptor‐2 (TLR2) is one of the best‐known pattern recognition receptors (PRR) of TLR families and is responsible for recognizing diverse molecules derived from pathogens, including LTA. It has been reported that TLR2 signaling activates the β‐catenin pathway to suppress chronic inflammation (Manoharan et al., 2014). Consistent with this, our present study found that LTA could activate downregulated β‐catenin signaling in macrophages during aging.

As a senescence‐regulating transcriptional factor, the FOXO1 expression changes with age. From 2 to 12 months of age, FOXO1 expression progressively decreased, whereas FOXO1 phosphorylation increased in the bone of aging mice (Rached et al., 2010). FOXO1 shuttles between the nucleus and the cytoplasm under physiological conditions. In the nucleus, FOXO1 was promoted to repress the p16/p53 pathway to prevent cell cycle arrest and maintain bone homeostasis (Rached et al., 2010). Under aging circumstances, phosphorylated FOXO1 was significantly increased, with reduced nuclear translocation of FOXO1 (Chen et al., 2019). The evidence above was consistent with our findings that FOXO1 signaling was downregulated and deactivated with aging, accompanied by increased cytoplasm and decreased nuclear translocation. FOXO1 phosphorylation results in disrupted interactions between the FOXO1 protein and its target DNA and leads to the translocation of the FOXO1 protein from the nucleus to the cytoplasm, thus suppressing FOXO1‐dependent transcription (Peng et al., 2020). Then, phosphorylated FOXO1 can be degraded through ubiquitination (Xing et al., 2018). In the present study, FOXO1 was phosphorylated under senescence circumstance. Therefore, the total p‐FOXO1 level increased and the total FOXO1 level decreased. Besides, the nucleus FOXO1 protein translocated to cytoplasm because of the phosphorylation, resulting in the upregulation of cytoplasm FOXO1 and downregulation of nucleus FOXO1. We further demonstrated LTA upregulated and activated FOXO1 signaling in macrophages, which was blocked by β‐catenin inhibitor, indicating that LTA could stimulate the FOXO1 signaling through activating the β‐catenin, which was in line with a recent study that increased β‐catenin upregulated FOXO1 and downregulated FOXO1 deactivation via phosphorylation, as well as lessened level of pro‐inflammatory cytokine in BMDMs (Yang et al., 2019).

Oxidative stress by excessive ROS, mainly represented by the H_2_O_2_ in cells, is one of the main causes of cellular senescence and can adversely affect bone homeostasis, leading to osteopenia (Kim et al., 2022). Therefore, we introduced the H_2_O_2_‐induced cellular senescence model to mimic the age‐related mice bone marrow microenvironment that BMDMs existed in. mTOR is regarded as a main driver of aging and depletion of mTOR has been shown to extend life span (Harrison et al., 2009; Herranz et al., 2015; Liu & Sabatini, 2020). A recent study revealed that senescent joint cells, caused by increased oxidative stress, showed upregulation of mTOR, promoting the SASP by phosphorylating MK2, which stabilized mRNA transcripts encoding SASP factors (including IL‐1 and IL‐6) to drive further senescence (Coryell et al., 2021). Autophagy was also reported to be regulated by mTOR under H_2_O_2_‐induced BMSC senescence circumstance, as the H_2_O_2_ increased the expression of p‐mTOR and aging phenotype, and negatively regulated the autophagy process in BMSCs, which were restored by rapamycin treatment (Liu, Yuan, et al., 2021). REDD1, an endogenous mTOR inhibitor, is reduced in joint synovium, meniscus, and cartilage during aging (Alvarez‐Garcia et al., 2016, 2017). Coincidently, we found an age‐related decline in the expression of REDD1 and a rise in the expression of phosphorylated mTOR in macrophages. Our study further demonstrated that LTA mediated the upregulation of REDD1 and downregulation of p‐mTOR, which were reversed by blocking β‐catenin and FOXO1 signaling. Our data extend the current understanding that upregulated mTOR phosphorylation in aging macrophages is a result of the downregulation of the β‐catenin/FOXO1/REDD1 signaling pathway.

This study has two limitations. First, we did not verify the direct relationship between mTOR signaling and age‐related bone degeneration by blocking the mTOR signaling. The characteristics of mTOR in aging are well known and the phosphorylation of mTOR is harmful to skeletal development (Moriceau et al., 2010; Spencer et al., 2006). Accumulating evidence indicated that mTOR signaling was activated in osteoporosis, and inhibition of mTOR signaling attenuated the osteoporotic phenotype in mice (Qi et al., 2017). Mechanically, LRRc17 has been proven to cause bone marrow‐derived mesenchymal stem cells (BMSCs) senescence and impair mitophagy through activating mTOR signaling, resulting in reduced osteogenic differentiation potential and weakened inhibitory effect on osteoclast differentiation (Liu, Yuan, et al., 2021). Rapamycin and its derivatives/analogs, as the mTOR inhibitors, have been reported to inhibit osteoclast formation and activity in vitro and prevent oophorectomy bone loss by 60% in vivo by reducing osteoclast‐driven bone resorption (Wang, Zhang, et al., 2023). Thus, we did not repeat the tests in this study but focused more on the upstream of mTOR and REDD1, the endogenous mTOR inhibitor. The other limitation of this work is we did not specifically knock down the β‐catenin and FOXO1 signaling in macrophages to verify the role of macrophage‐specific β‐catenin/FOXO1 signaling in bone remodeling. It will be important in future work to assess the functional role of macrophage‐specific β‐catenin/FOXO1 signaling in controlling bone remodeling and generation.

## 5 CONCLUSION

In summary, our study demonstrates that downregulation of β‐catenin/FOXO1 signaling pathway in macrophages with aging may be one of the critical mechanisms that induce the macrophage senescence and bone degeneration, accompanied by downregulation of REDD1 and upregulation of p‐mTOR. LTA rescues the β‐catenin/FOXO1 signaling to prevent macrophage senescence and bone loss, which could be exacerbated by β‐catenin/FOXO1 inhibition. This study establishes the essential role of β‐catenin/FOXO1/REDD1 signaling pathway in bone degeneration during aging.

## AUTHOR CONTRIBUTIONS

G.L. and B.Y. conceived and designed the study. W.C. and Y.F. carried out most of the experiment. Z.L., M.H., Y.C., Y.H., and Q.L. performed several experiments. W.C. and Z.L. drafted sections of the manuscript. W.C. and Y.F. composed the figures in this manuscript. W.C., G.L., and B.Y. revised and approved the manuscript.

## FUNDING INFORMATION

This work was supported by The Major Program of National Natural Science Foundation of China (81830079, 82201732). We gratefully acknowledge all individuals who participated in the study.

## CONFLICT OF INTEREST STATEMENT

None of the authors has any potential financial conflict of interest related to this manuscript.

## Supporting information


Figures S1–S4

## Data Availability

The datasets of the present study are available from the corresponding authors on reasonable request.
