# Supplementary material for: Lipoteichoic acid restrains macrophage senescence via β‐catenin/FOXO1/REDD1 pathway in age‐related osteoporosis
Source: Aging Cell. 2023 Dec 21;23(3):e14072. doi: 10.1111/acel.14072 (PMC10928565; doi:10.1111/acel.14072)
Supplement: Supplementary file 1 — Figures S1–S4 [file ACEL-23-e14072-s001.pdf]

Supplemental figure 1

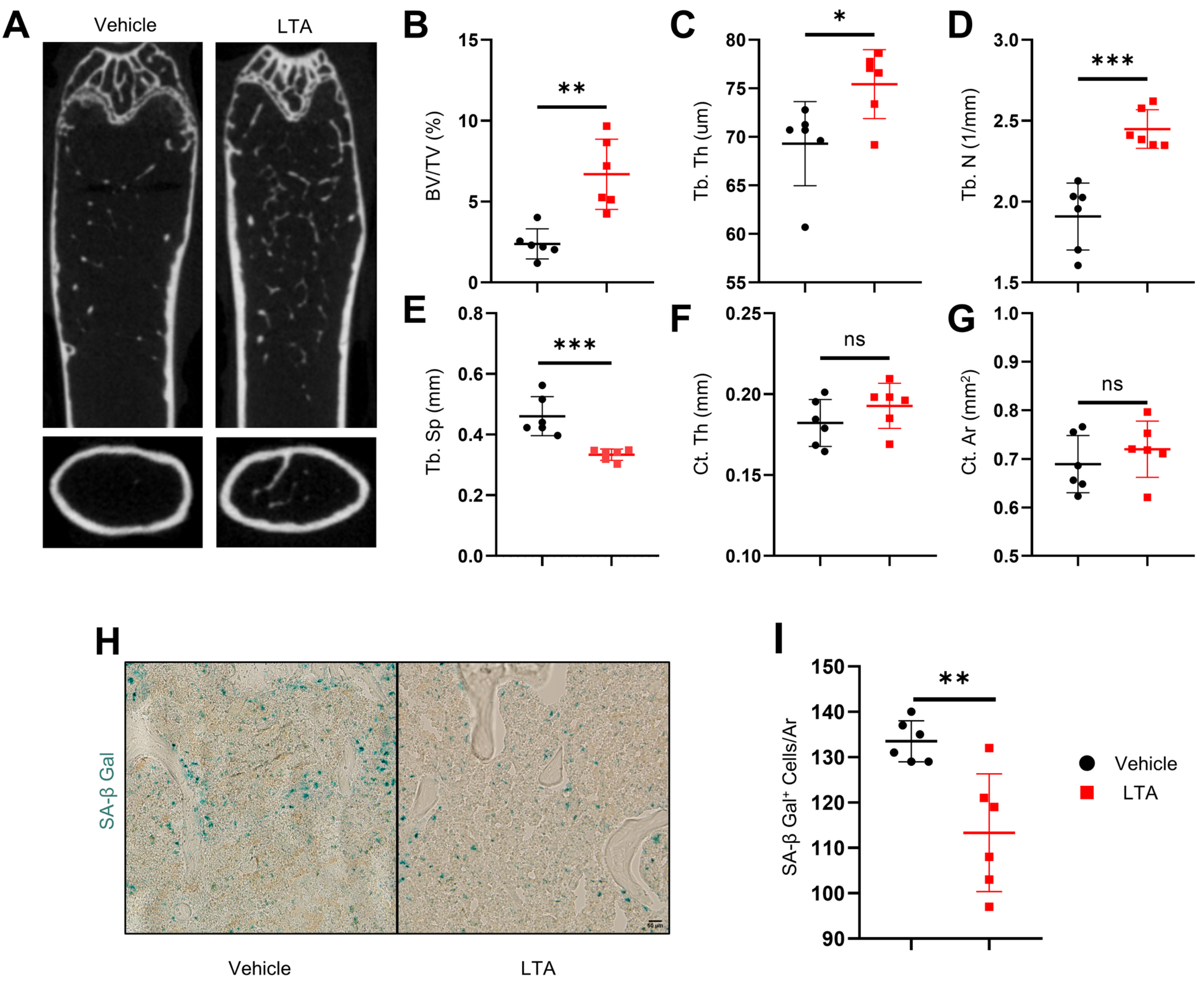

Figure S1: LTA ameliorates age-related osteoporosis and attenuates intramedullary cellular senescence. Representative μCT images **(A)** of longitudinal sections and cross-sections of femoral bone from vehicle- and LTA-treated 12-month-old mice. Quantitative analyses of trabecular bone volume fraction (BV/TV) **(B)**, trabecular thickness (Tb. Th) **(C)**, trabecular number (Tb. N) **(D)**, trabecular separation (Tb. Sp) **(E)**, cortical bone thickness (Ct. Th) **(F)** and cortical bone area (Ct. Ar) **(G)**. n = 6/group. Representative images **(H)** and quantification **(I)** of SA-β-Gal staining of femoral sections from vehicle- and LTA-treated 12-month-old mice. n = 6/group. Scale bars, 50μm. *P < 0.05, **P < 0.01, ***P < 0.001. Data are presented as mean ± SD. 2-tailed Student's t-test was used.

Supplemental figure 2

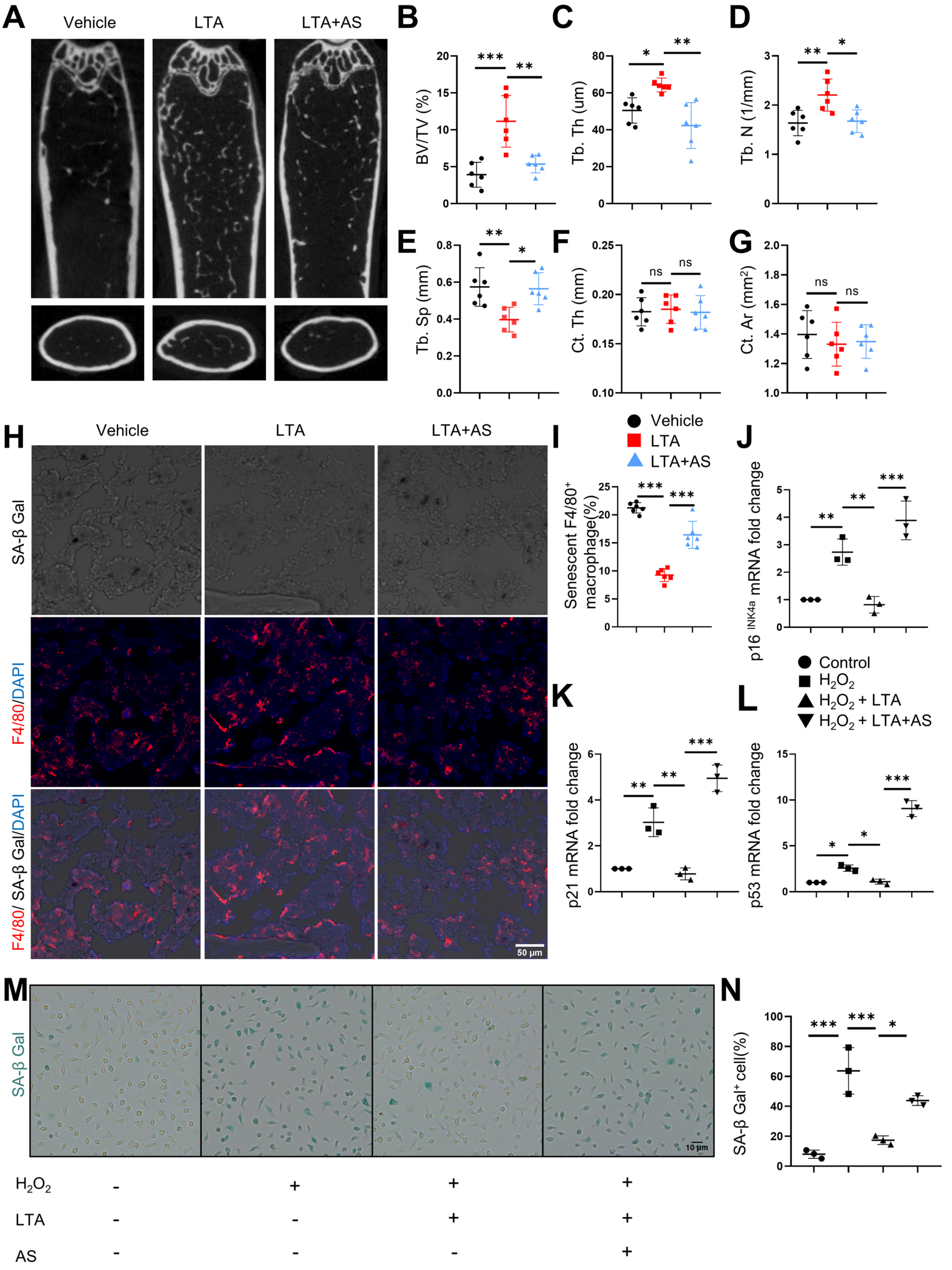

Figure S2: Blocking FOXO1 deteriorates bone degeneration and accelerates macrophage senescence. 12-month-old mice were treated with LTA together with AS. Representative μCT images **(A)** of longitudinal sections and cross-sections of femoral bone and quantitative analyses of trabecular bone volume fraction (BV/TV) **(B)**, trabecular thickness (Tb. Th) **(C)**, trabecular number (Tb. N) **(D)**, trabecular separation (Tb. Sp) **(E)**, cortical bone thickness (Ct. Th) **(F)** and cortical bone area (Ct. Ar) **(G)**; n = 6/group. Representative images **(H)** of SA-β-Gal staining (gray) and immunofluorescence staining of F4/80 (red) of femoral sections and quantification **(I)** of the number of senescent F4/80$^+$ macrophages in the femoral bone of 3 groups of mice. n = 6/group. Scale bars, 50 μm. H_2O_2-induced senescent BMDMs were treated with LTA together with AS. Quantitative real-time PCR analysis of p16^{INK4a} **(J)**, p21 **(K)**, and p53 **(L)** for 4 groups of BMDMs. n = 3/group. Representative images of SA-β-Gal staining (blue) **(M)** and quantification **(N)** of the percentage of SA-β-Gal$^+$ BMDMs for 4 groups of BMDMs. n = 3/group. Scale bars, 10 μm. AS: AS1842856. *$P < 0.05$, **$P < 0.01$, ***$P < 0.001$. Data are presented as mean ± SD. One-way ANOVA with Tukey's test.

Supplemental figure 3

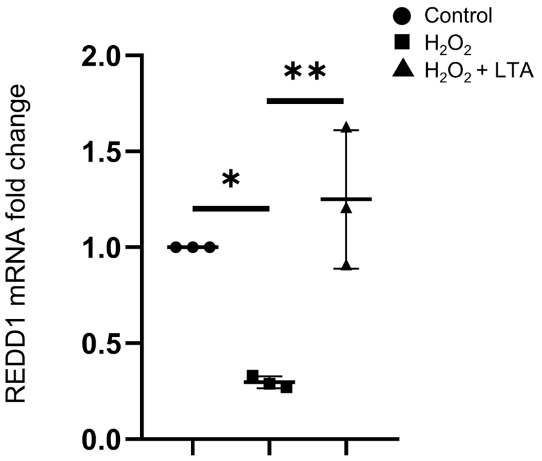

Figure S3: LTA restored the downregulation of REDD1 mRNA level caused by H_2O_2. Quantitative real-time PCR analysis of REDD1 for 3 groups BMDMs. n = 3/group. *P < 0.05, **P < 0.01. Data are presented as mean ± SD. One-way ANOVA with Tukey's test.

Supplemental figure 4

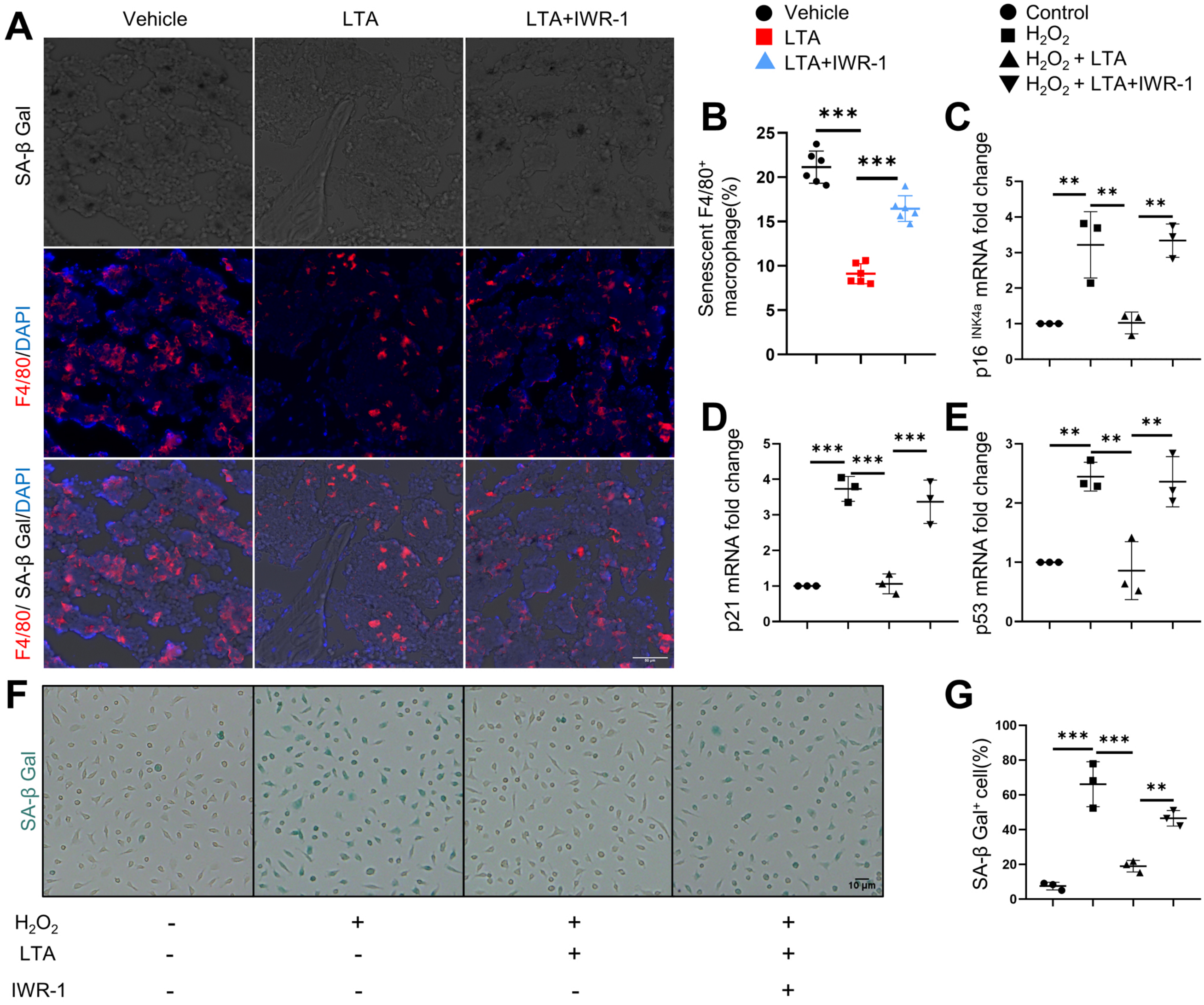

Figure S4: Blocking β-catenin boosts macrophage senescence. Representative images **(A)** of SA-β-Gal staining (gray) and immunofluorescence staining of F4/80 (red) of femoral sections and quantification **(B)** of the number of senescent F4/80$^+$ macrophages in the femoral bone of 3 groups of 12-month-old mice. n = 6/group. Scale bars, 50 μm. H_2O_2-induced senescent BMDMs were treated with LTA together with IWR-1. Quantitative real-time PCR analysis of p16^{INK4a} **(C)**, p21 **(D)**, and p53 **(E)** for 4 groups BMDMs. n = 3/group. Representative images of SA-β-Gal staining (blue) **(F)** and quantification **(G)** of the percentage of SA-β-Gal$^+$ BMDMs for 4 groups of BMDMs. n = 3/group. Scale bars, 10 μm. *P < 0.05, **P < 0.01, ***P < 0.001. Data are presented as mean ± SD. One-way ANOVA with Tukey's test.
